# The role of growth hormone in metastasis and angiogenesis of breast cancer

**DOI:** 10.1042/BSR20253516

**Published:** 2026-02-16

**Authors:** Tamanna Alam, Mingyang Hu, Rivka L. Isaacson

**Affiliations:** Department of Chemistry, King’s College London, Britannia House, 7 Trinity Street, London, SE1 1DB, U.K.

**Keywords:** angiogenesis, breast cancers, growth hormones, insulin-like growth factor, metastasis

## Abstract

Breast cancer is a complex disease which has many factors affecting its progression and metastasis. Although steroid hormones, especially oestrogen, are most commonly associated with breast cancer, growth hormone (GH) also plays a substantial role in its development and spread via the activation of downstream signalling pathways and the regulation of growth factors such as insulin-like growth factor-1 (IGF-1) and the vascular endothelial growth factor (VEGF). Breast cancer patients usually have elevated levels of GH and IGF-1 in their circulation. Growth hormone receptor (GHR) signalling enhances migratory ability of tumour cells and excess IGF-1 production promotes angiogenesis. Gaining a full understanding of the mechanisms behind GH and breast cancer will allow researchers to develop more therapeutics to treat this devastating disease.

## Introduction

Breast cancer is a multifaceted neoplastic disease characterised by the proliferation of cells in the breast tissue to form a tumour. In 2022, approximately 2.3 million women were diagnosed with breast cancer, resulting in 670,000 deaths globally [1]. Breast cancer is one of the most commonly diagnosed cancers in the U.K. [2].

The strongest risk factor for breast cancer is being female; however, 0.5–1% of cases occur in males [1]. The risk of developing breast cancer increases with age; although it is possible to develop breast cancer at any age, the majority of cases are diagnosed in women over the age of 50 [2]. A family history of breast cancer increases one's risk of developing it [2]. *BRCA1* and *BRCA2* genes encode BRCA1 and BRCA2 proteins, which are important for various cellular processes including the maintenance of genomic stability, facilitating DNA repair and cell cycle regulation [3]. Mutations in these genes can impair their normal functions, which is associated with hereditary breast cancer [2]. Hormonal factors such as premature onset of menarche (the first menstrual cycle), delayed menopause, or increased levels of oestrogen also increase the risk of breast cancer [4].

Breast cancer can be broadly separated into two categories: hormone dependent and hormone independent [5]. Whilst the sex hormones, progesterone, and oestrogen, are most frequently associated with the disease, research indicates that growth hormone (GH) also plays a vital role in the development of breast cancer [4,6]. This review aims to explore the lesser-known, pivotal role that GH plays in the progression of breast cancer through angiogenesis (growth of blood vessels) and metastasis (spread of cancer).

## The structure and function of growth hormone

**Figure 1:**
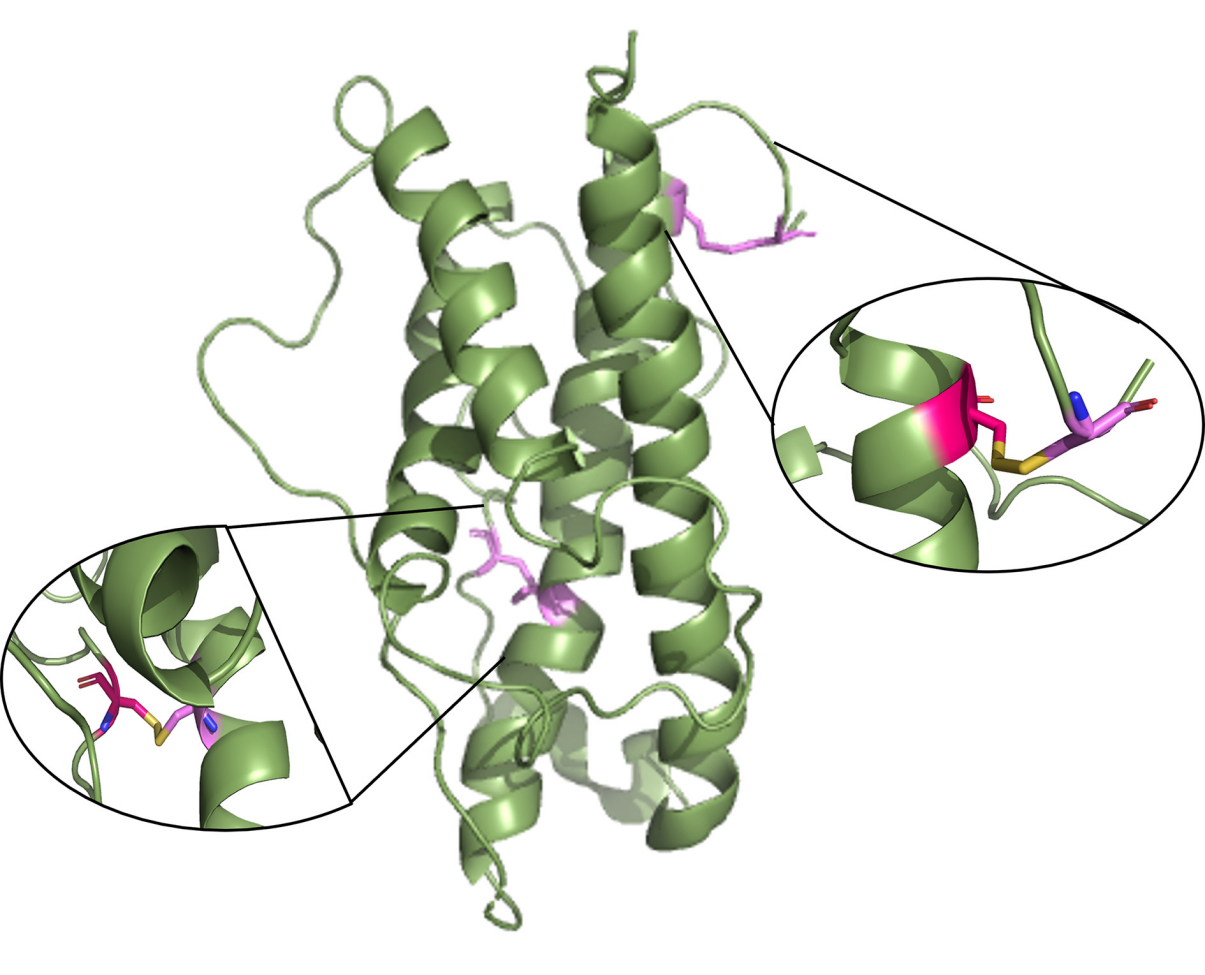
Structure of somatotrophin or human growth hormone GH is composed of four helices. Two disulphide bridges are indicated in violet. Specific residues forming these disulphide bridges include Cys-53 and Cys-165 (left zoom), and Cys-182 and Cys-189 (right zoom). Figure made using Protein Data Bank (PDB) Accession number: 1HGU.

Growth hormone (somatotrophin or hGH, in its human form) is a pituitary hormone that promotes growth of tissues and bones, and cell production and regeneration in both humans and animals [7]. It is encoded by the *GH1* gene and is a polypeptide consisting of 191 amino acids [8]. Its primary structure dictates its three-dimensional fold which forms a characteristic four-helical bundle protein that has a molecular mass of 22 kDa (Figure 1) [9]. Its structural motif involves two helices that are parallel to each other and antiparallel to the other two helices; each pair of helices is connected by long loops [9].

GH exhibits two disulphide bridges crucial for maintaining its structural integrity and functionality [9]. One disulphide bridge is located at a region linking the N- and C- termini (between Cys-53 and Cys-165) whilst the other occurs only at the C-terminal region (between Cys-182 and Cys-189) (Figure 1). Both disulphide bridges contribute to the stabilisation of the overall protein fold [10]. The C-terminal disulfide bridge is evolutionally conserved and essential for the binding of GH to its receptor [10].

Moreover, GH harbours two zinc-binding sites between His-18, His-21, and Glu-174 which are essential for dimerisation and secretory granule biogenesis [11]. Secretory granule biogenesis is the process by which hormones and enzymes are stored in membrane-bound vesicles called secretory granules [12]. This is crucial for the storage and rapid release of GH. During human development, levels of GH rise gradually in childhood and peak during puberty to orchestrate various growth processes [13].

The release of GH from the pituitary gland is triggered by the secretion of growth hormone releasing hormone (GHRH) from the hypothalamus [13]. GH exerts its growth effects by stimulating the liver to release insulin-like growth factor-1 (IGF-1) and inducing the degradation of glycogen into glucose to provide energy for cellular growth and metabolism [14]. IGF-1 is the primary mediator for the biological activity of GH [14]. The growth effects owing to this are an increase in the uptake of amino acids, enhancements to cellular proliferation, angiogenesis (growth of blood vessels), elongation of bones, and reduction of apoptosis (cell death) [14]. The biological actions of GH are mediated through its interaction with growth hormone receptor (GHR), which initiates downstream signalling pathways required for growth [6].

## Growth hormone in mammary gland development

The endocrine system intricately controls the development of the mammary gland, a process that is completed at puberty. The mammary gland undergoes several dynamic changes throughout a woman’s life [6]. Recent studies have shed light on the interactions between progesterone and GH within the mammary gland microenvironment. GH is secreted locally by normal mouse breast cells upon the stimulation of progesterone [15]. The local production of GH influences the activity of mammary stem cells via interactions with other components of the endocrine system. GH was found to enhance the rate of proliferation of mammary stem cells leading to the expansion of the stem cell population needed for continued development of the mammary gland [15].

At the molecular level, the activation of GH/GHR is also implicated in the paracrine signalling system, whereby paracrine cells produce signalling molecules, like hormones, to be received by nearby cells [16]. The signalling molecules are typically released into the extracellular fluid in order to reach target cells in the immediate vicinity of the secreting cell [16]. GH expresses proliferation signals initiated by steroid hormones (e.g. progesterone) to mammary stem cells [15]. These findings propose that GH plays a pivotal role in the development and maintenance of the mammary gland by influencing cellular proliferation and by engaging in interactions with the endocrine system. It also suggests the possibility that GH secretion, in response to progesterone stimulation, may occur regularly in adulthood when GH secretion from the pituitary gland declines [16]. This process may promote an increase of mammary stem cells during certain developmental windows, or hormonal phases, which could conceivably contribute to the uncontrolled proliferation of breast cells that ultimately leads to breast cancer development.

Whilst these findings offer details about the biological interaction of GH and progesterone during mammary gland development and post-development secretion, it is important to acknowledge the potential effects of confounding factors, such as the presence of other hormones and growth factors within the mammary gland that may influence GH signalling. In order to seamlessly translate these findings to breast cancer research, further studies must consider and control for these factors. Since these studies were conducted in mice, extrapolating the findings to human biology should be carried out with caution. The clinical relevance to breast cancer carcinogenesis and any treatment outcomes arising from this would require further investigation.

## Growth hormone receptor

**Figure 2:**
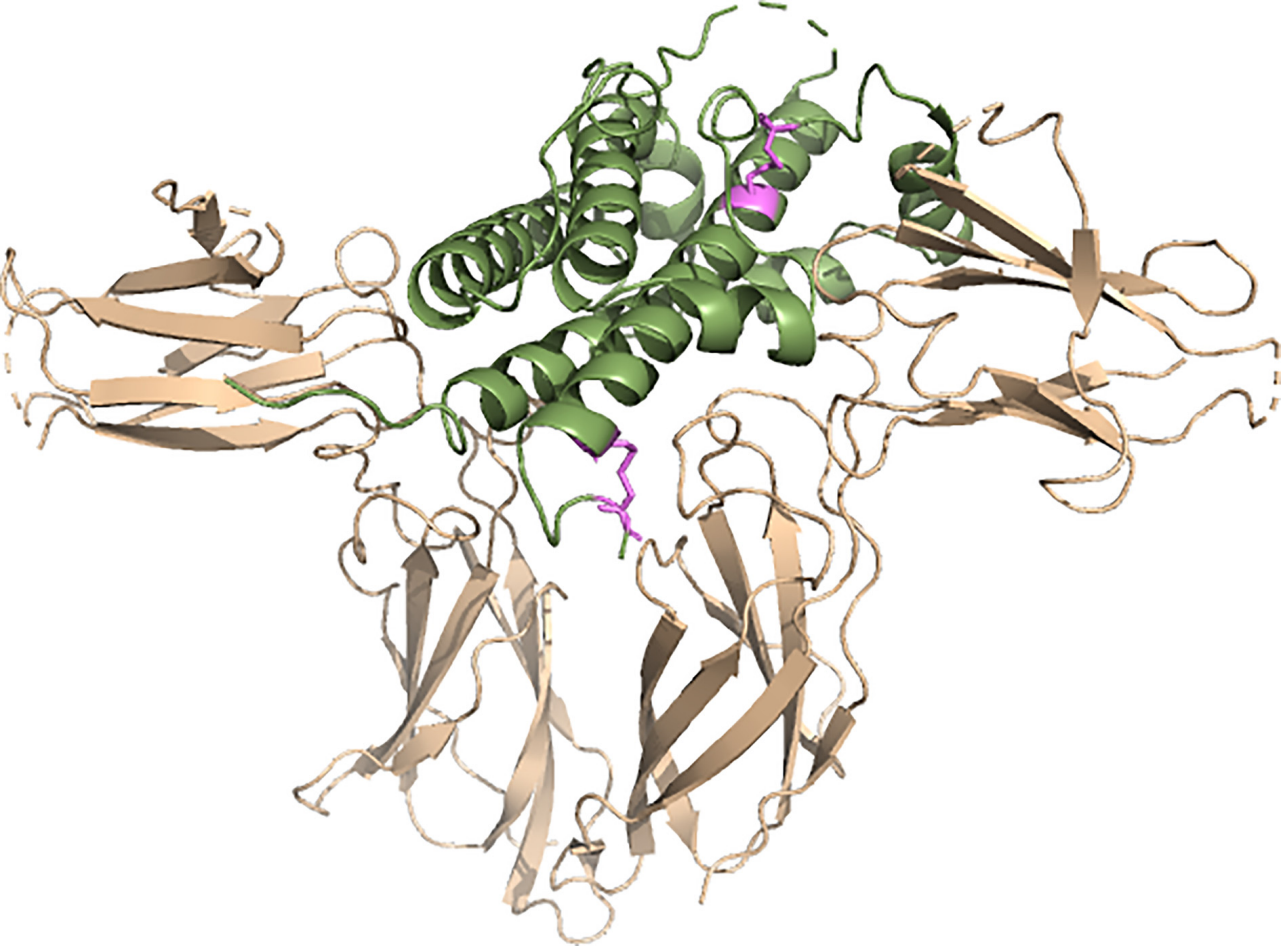
Bound human growth hormone receptor complex structure Structure of GHR (cream) and GH (green) in complex (created using PDB accession code: 3HHR^9^). GHR is a homodimeric receptor that, once bound to GH, activates signal transduction pathways which are vital for growth.

Growth hormone receptor is a homodimeric transmembrane protein consisting of 620 amino acids and is the protein responsible for orchestrating the physiological functions of GH (Figure 2) [10]. Structurally, GHR belongs to the Type I cytokine receptor family, characterised by its extracellular ligand-binding domain, transmembrane domain, and intracellular signalling domain [17]. Cytokine receptors bind to cytokines, small membrane-bound glycoproteins that take part in cell signalling [18]. There is some uncertainty about how GH and GHR interact. Earlier work suggested that GH initially binds to the monomeric receptor, which generates a conformational change in the intracellular domain of GHR, so that it interacts with a second identical receptor inducing homodimerisation and activation [17]. Later studies reported that GH can bind to pre-dimerised GHR using its two asymmetric binding sites, which differ in binding affinity, to create a trimolecular complex that initiates downstream signalling pathways [17,19,20]. The GH/GHR interaction activates various signal transduction pathways vital for cell growth including Janus Kinase/signal transducer and activator of transcription pathway (JAK2/STAT5a/b) [10]. The activation of JAK2 phosphorylates multiple tyrosine residues on the intracellular domain of the receptor [17]. GHRs are found on the cell surfaces of various tissues and organs throughout the body, including liver, muscle, kidney, and in early embryonic and foetal tissue [17].

Interestingly, an interaction has also been reported between GHR and the co-chaperone small glutamine-rich, tetratricopeptide repeat protein alpha (SGTA) [21,22], which has a known role in breast and prostate cancers through its association with steroid hormone receptor biogenesis [23,24] and Rec, a cancer-elevated human protein of retroviral origin [23].

Structural studies, employing techniques such as X-ray crystallography and molecular modelling, have provided valuable insights into the molecular architecture of the GH/GHR complex [25,26]. By elucidating the intricate network of interactions between GH and GHR, the importance of specific amino acid residues and structural motifs in mediating receptor-ligand action, receptor dimerization, and signal transduction, is highlighted. Thus, by investigating the molecular mechanisms underlying the GH/GHR interaction, deeper insight can be gained into the biological actions of GH and its role in regulating cellular processes such as growth, metabolism, and development. Moreover, these insights can pave the way for the development of novel therapeutics targeting the GH/GHR signalling axis for the treatment of growth disorders and cancers.

## Metastasis

Metastasis is a critical step in cancer progression whereby cancer cells spread to other parts of the body through blood and the lymphatic system to form secondary tumours [27]. The most likely areas for secondary tumours are the lungs, bones, liver, and the brain. Once breast cancer has become metastatic there is no cure, however treatment options become available to prolong survival; metastasis is the leading cause of death for patients with a breast cancer diagnosis [27].

Metastasis is a complex event in tumorigenesis [28]; 20–30% of breast cancers become metastatic. Epithelial-to-mesenchymal transition (EMT) is the ability of an epithelial cell to transition into a mesenchymal cell during embryogenesis, tissue repair, or tumorigenesis [29]. A mesenchymal cell is characterised by its ability to differentiate into different types of cells. EMT plays an important role in metastasis as it enables normal epithelial cells to acquire mesenchymal characteristics and attain a ‘plastic phenotype’ which then leads to tumours becoming invasive and metastatic. Research has indicated that GH can induce EMT and therefore promote metastasis [29].

The steps in metastasis include escape of cancer cells from the primary tumour, intravasation, survival maintenance, extravasation, and outgrowth [28]. Cancer cells attain a ‘plastic phenotype’ to achieve an invasive state – this process is called invasion and is a pre-requisite in metastasis which allows cancer cells to escape the site of the primary tumour [30]. Researchers have explored how harnessing this plasticity through genetic modifications can impede a tumour’s ability to metastasise [31]. However, this study does not account for the immunosuppressive ability of tumours, which is common in breast cancers, since the study was conducted in an environment without T cells [32]. Thus, further testing in a more realistic cancerous environment, may be necessary to take this forward therapeutically. Furthermore, carrying out genetic modification treatments on tumours within patients may be challenging to execute.

During intravasation, tumour cells penetrate the basement membrane of the tumour and pierce nearby blood vessels to enter the blood circulation [33]. This process leads to the planting of potential metastatic seeds in other areas of the body. The process of intravasation requires degradation of the basement membrane, blood vessels, and the extracellular matrix [34]. The degradation of the extracellular matrix is critical for the production of vascular endothelial growth factor (VEGF) which promotes tumour angiogenesis and extravasation of tumour cells [35].

Cancer cells that are headed towards blood vessels have a very short half-life. These cells are exposed to a high amount of stress due to loss of adhesion from the primary site and exposure to destruction signals from the body’s immune system [28]. It is estimated that less than 0.02% of cells that have left the primary tumour site will form secondary tumours [31]. The low fractions of cells that do survive have a highly effective metastatic fate.

Extravasation involves tumour cells leaking from the blood vessels into tissues [33]. Platelets are important for facilitating metastasis during extravasation. Platelets shield cancer cells, promoting blood clotting and weakening the endothelial barrier, which allows for an easier pathway for tumour cells to penetrate the blood vessels and enter circulation [28]. Once the cancer cells reach a site where they can form a secondary tumour, they can either begin to proliferate or enter a state of dormancy [28,36]. The proliferative cells can go on to form secondary tumours; however, the dormant cells can remain dormant for years until they become detectable cancers [36].

A study investigated the process of dormant cancer cells becoming proliferative cancer cells using microphysiological models to mimic breast cancer metastases in the liver. Dormant cells receive various signals from the surrounding environment to transition to the proliferative state; this may be from cytokines, growth factors such as VEGF which stimulate angiogenesis, or interactions with the immune system [36].

## GHR and metastasis

GHR signalling is known to play a role in the development of breast tumours and metastasis through promoting the EMT pathway [29]. Although the exact mechanism of how GHR signalling takes part in metastasis is not known, researchers investigated this by inhibiting GHRs and therefore the action of GH in breast cancer cell lines [15,37,38]. They first analysed a variety of breast tissues and found that GHR is overexpressed in breast cancer tissues and cells compared with normal tissues and cells in mice [37]. Next they silenced GHR via small hairpin RNA (shRNA), a method used to inhibit specific genes, in human breast cancer cells and then transplanted these cells into mice. Migratory ability of the cells was used as an indicator of metastatic potential. Compared with the control group, migratory ability was significantly reduced in cells where GHR was silenced, thus indicating that the silencing of GHR and therefore inhibition of GH reduces the cells’ ability to migrate, which is a key component of metastasis [37].

Whilst this study offers important observations into the role of GHR in breast cancer metastasis, it focused primarily on a subtype of breast cancer, oestrogen-receptor negative. It is possible that GHR signalling influences the cancer in different ways in oestrogen-receptor positive tumours considering the prominent role of oestrogen within these tumours. Furthermore, the study relies on cell culture environments; it would be beneficial for future research to explore the broader implications of GHR modulation in patient-derived samples or clinical trials. This would strengthen the relevance of the findings. Nevertheless, the study assesses the effects of GHR inhibition on breast cancer metastasis and the experimental approaches chosen by researchers provide valuable insights into the action of GH in metastasis.

Another study conducted *in vivo* experiments using patient-derived xenograft models to exhibit the effect of GH on tumour growth. Upon the inhibition of GHR signalling, a significant reduction of tumour growth was seen indicating the potential impact of GH in influencing the development and progression of breast tumours [15]. However, this study was only conducted over a 2-week period, potentially overlooking the effects and long-term consequences of GH inhibition on tumours. Thus, a longer research period is needed in order to translate these findings into a clinical setting.

**Figure 3:**
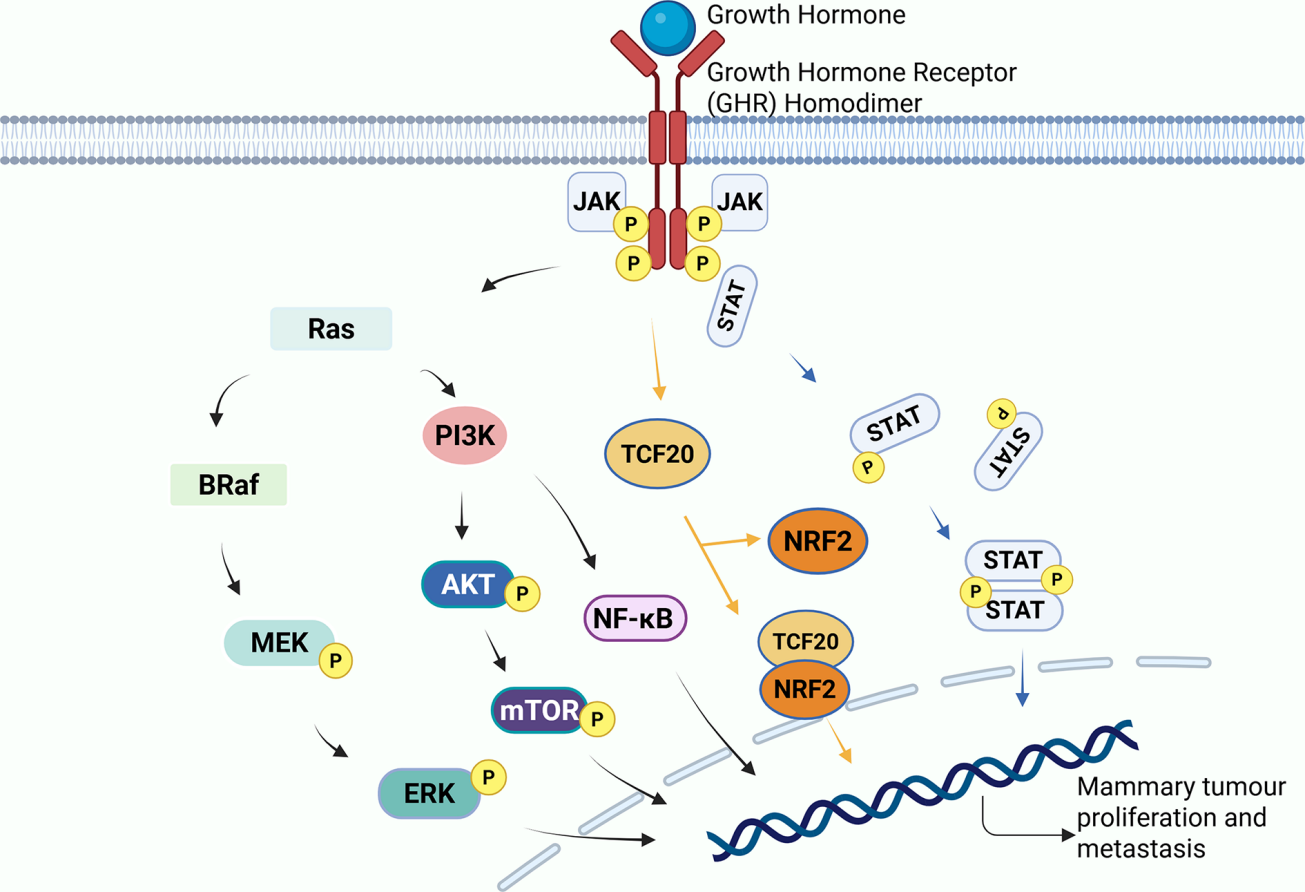
GH/GHR-driven signalling pathways that promote breast cancer progression Growth hormone (GH) binding to the growth hormone receptor (GHR) activates several signalling cascades that support tumour growth and metastasis. GHR stimulates the JAK2–STAT5 pathway, the PI3K–AKT–mTOR axis, NF-κB, the TCF20-NRF2 and the Ras–RAF–MEK–ERK cascades, each contributing to increased proliferation and metastasis.

Multiple studies have examined the potential molecular pathways through which GHR promotes breast cancer cell proliferation and metastasis (as summarised in Figure 3). As previously reported, shRNA-mediated knockdown of GHR markedly reduced the migratory ability of breast cancer cells [37]. The decrease in viability was accompanied by diminished phosphorylation of AKT and mTOR, indicating inhibition of the AKT/mTOR signalling axis. In the well-established JAK2/STAT5a/b hormone signalling pathway, the reduction of phosphorylated JAK2, STAT3, and STAT5a/b were also observed in GHR silenced cells [37]. Together, these findings demonstrate that GHR supports breast cancer cell survival and metastasis at least in part through activation of both the JAK2/STAT and AKT/mTOR pathways.

A separate study identified an additional mechanism by which GHR contributes to mammary tumour progression. In MDA-MB-231 and MCF-7 cells, siRNA-mediated GHR silencing resulted in reduced phosphorylation of BRAF, MEK and ERK across both cell lines, alongside decreases in tumour volume and weight in xenograft models [39]. Given that activation of the BRAF/MEK/ERK cascade facilitates EMT [40], a key driver of cancer cell metastasis, its inhibition following GHR silencing may further impede metastatic potential. However, the molecular subtype of the breast cancer cell lines used was not specified, and additional contextual information would improve interpretation of these results. Moreover, several studies have established the link between GH/GHR signalling and activation of the nuclear factor -kappa B (NF-κB) pathway, and consequent promotion of mammary carcinoma metastasis. In MCF-7 cells engineered to express human GH (hGH-MCF-7), the NF-Κb activity was substantially increased compared with that in MUT-MCF-7 controls, leading to marked reductions in E-cadherin and P-cadherin expression [41]. Given that loss of E-cadherin is a defining early step in EMT [42], these observations position NF-κB activation as a central mediator of GH-driven migration. Intriguingly, pharmacological intervention has revealed cross-talk between the PI3K–AKT pathway and NF-κB activation. Inhibition of PI3K with LY294002, a PI3K inhibitor, suppressed phosphorylation of PI3K and AKT and simultaneously reduced levels of phosphorylated IκBα, total NF-κB p65 and phosphorylated NF-κB p65 [43]. These data imply that NF-κB activation relies, at least partially, on upstream PI3K–AKT signalling, supporting the concept of a PI3K–AKT–NF-κB axis downstream of GH/GHR. However, the affinity of LY294002 to PI3K was not directly evaluated in this study, leaving the possibility of off-target effects e.g. binding to an undetected protein which could also contribute to the decrease in the level of NF-κB p65, and p-NF-κB p65. Therefore, future work incorporating more selective PI3K inhibitors or genetic approaches will be important for validating the mechanistic relationship between these pathways.

A very recent study revealed that elevated endocrine GH promotes the growth and spread of triple-negative breast cancer (TNBC) in acromegaly mouse models by up-regulating antioxidant genes [38]. Using this acromegaly model, the researchers examined how excess GH influences TNBC progression and metastasis. They found that elevated GH activates transcription factor 20 (TCF20), a nuclear protein, which in turn drives the transcription of nuclear factor erythroid 2-related factor 2 (NRF2) and its target genes, facilitating TNBC metastasis. Blocking the GH receptor (GHR) and TCF20 activity with a GHR antagonist or gene knockdown via small-interfering RNA reduced both tumour size and metastasis, indicating that excess endocrine GH enhances TCF20/NRF2 pathways in TNBC, promoting lung metastasis [38]. As the work used a TNBC model, it expands on the earlier study which primarily examined oestrogen receptor-negative breast cancer [37], broadening the understanding of GH/GHR's role across different breast cancer subtypes. In addition, this finding underscores the potential of targeting GH–TCF20–NRF2 signalling pathways as a therapeutic strategy against breast cancer metastasis, especially in hormone-independent subtypes such as TNBC.

In conclusion, these findings illustrate that GH/GHR signalling drives breast cancer progression and metastasis through multiple, interconnected pathways, including JAK2/STAT, MEK-ERK, PI3K–AKT, NF-κB and TCF20-NRF. Furthermore, the need for further mechanistic refinement is highlighted across defined molecular subtypes.

## Insulin-like growth factor (IGF-1)

**Figure 4:**
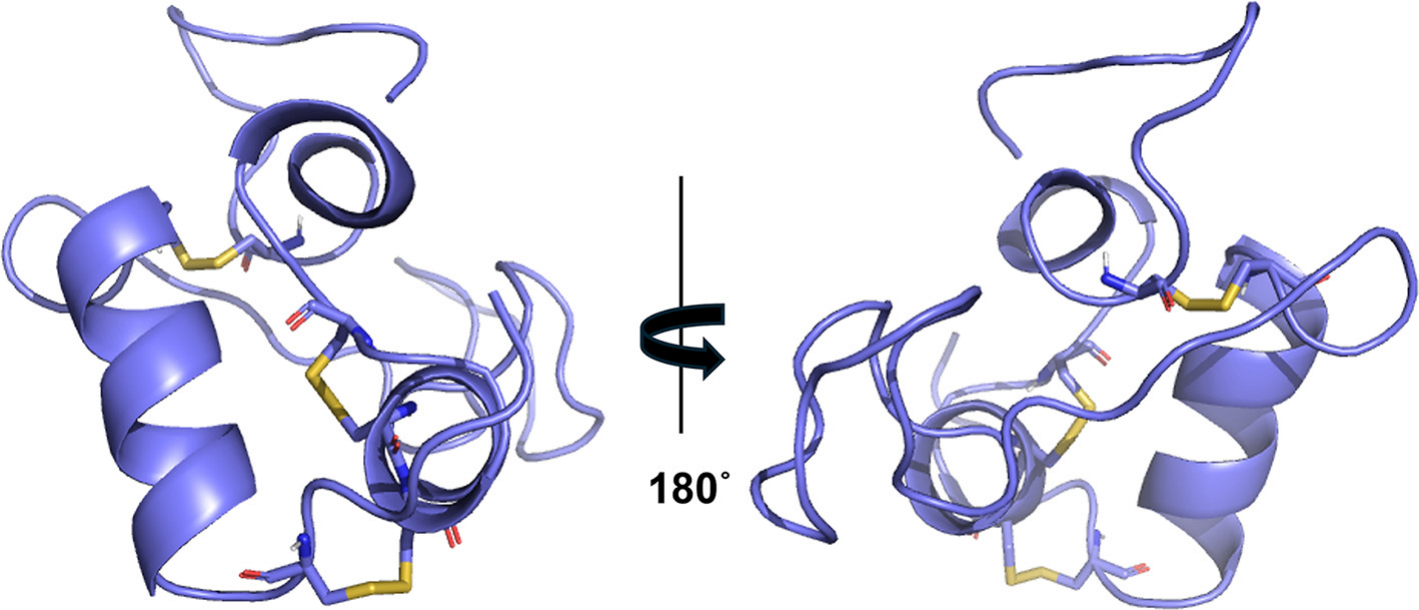
Structure of IGF-1 Created using PDB accession code: 1B9G. Disulphide bonds are indicated in yellow.

IGF-1 is a small peptide consisting of 70 amino acids in a single chain with three intramolecular disulfide bridges located between Cys-18/Cys-48, Cys-6/Cys-35 and Cys-34/Cys-39 (Figure 4) [44]. These disulfide bridges act as ‘safety pins’, keeping the protein small and compact, and are important for the binding of IGF-1 to its receptors and for storage in the liver and biological activity [44]. IGF-1 is produced throughout life at varying levels and is involved in the proliferation and function of nearly every cell, tissue and organ in our bodies. IGF-1 circulation concentration is lowest at infancy and old age and is at its highest during growth spurts at puberty. Insulin levels, genes, sex, age, stress, nutritional levels, and body mass index are other factors that can cause IGF-1 levels to vary. IGF-1 is the primary mediator of the physiological effects of GH [44]. Overexpression of IGF-1 enhances uncontrolled cellular proliferation which leads to tumour growth and angiogenesis [45]. Angiogenesis refers to the ability of tumours to establish their own blood vessels for a sufficient blood supply to the tumour for its continued growth and survival [45].

IGF-1 plays a significant role in the regulation of growth plates in bones [46]. Growth plates are areas of cartilage near the ends of long bones where growth occurs, IGF-1 is a key mediator of the growth-promoting effects of GH on these plates [46]. In some research studies, height was used as a biomarker of the GH/IGF-1 interaction to calculate cancer risk [39]. Results have indicated that individuals taller than 170 cm in height have a higher risk of developing different types of cancer as well as breast cancer compared to their counterparts in the control group who were shorter than 160 cm. They found a 20% higher risk for prostate cancer, a 20–60% higher risk for colorectal cancer, and a 22% higher risk for breast cancer [39]. These results suggest a strong correlation between GH, and therefore IGF-1 production, and cancer risk.

Another study investigated the sera of around 4,000 breast cancer patients and found that IGF-1 levels (including IGF-1 and IGF-1 binding proteins) were raised in the sera of breast cancer patients compared with a control group [47]. The study also analysed the sera of non-breast cancer patients and split them into five groups based on IGF-1 circulation concentration. Those in the group with higher IGF-1 levels (highest fifth) exhibited specific biological characteristics; they were younger, taller, and therefore, had a greater risk of developing breast cancer compared with the lowest fifth [47]. Thus, researchers established a probable causal relationship between IGF-1 concentration and breast cancer risk. These findings contribute to the understanding between IGF-1 and breast cancer risk and demonstrate how future research should investigate interventions to target the IGF-1 pathway and reduce the risk of breast tumorigenesis. Younger individuals typically have higher IGF-1 levels due to pubertal growth spurts and researchers have not published the specific ages of these participants. The conclusions made by the researchers regarding IGF-1 and breast cancer risk may be influenced by the age distribution of the sample.

IGF-1, IGF-1 receptors and IGF-binding proteins make up the IGF-1 system which also plays a substantial role in the development of the mammary gland [48]. The mammary gland is made up of epithelial tissue forming ducts and lobules [49]. At birth, it consists only of a few small ducts that undergo ductal morphogenesis until puberty. At puberty, a ‘ductal tree’, the morphing of branched structures in the gland, is formed and this process is regulated by GH and IGF-1 [49].

## IGF-1 and angiogenesis

Excess IGF-1 production, stimulated by GH, has been shown to increase angiogenic abilities of breast tumours [45]. The process of angiogenesis begins with the activation of endothelial cells, which are the cells that line blood vessels. Upon activation they proliferate and migrate towards a site of new vessel formation. Once the endothelial cells have reached the secondary site, they begin to form a tube-like structure that will eventually become a new blood vessel [50]. This process is regulated by several different signalling pathways, including the VEGF pathway, which is an important regulator of angiogenesis and metastasis in breast cancer [44]. When IGF-1 binds with insulin-like binding proteins it increases activity and bioavailability, further stimulating angiogenic effects of breast tumours [50].

A study conducted on collagen gel co-culture models using endothelial cells observed the process of angiogenesis upon exposure to IGF-1 [51]. Collagen is the most abundant protein found in the extracellular fluid of tissues; it provides structural support to cells and regulates cell-to-cell adhesion [52]. Researchers employed this technique to mimic the natural environment of a cell and applied confocal laser scanning microscopy to visualise the effect of IGF-1 on angiogenesis [51]. The study found that IGF-1 treatment promoted the formation of new blood vessels. Microscopic observations displayed a larger number of vessel-like structures between IGF-1 treated cells compared with a co-culture where cells were not treated with IGF-1. Quantitative analysis of vessel lengths and widths indicated a positive relationship between the effect of IGF-1 and angiogenesis compared to the co-culture group [51].

A study mentioned previously [45] found that IGF-1 levels are raised in breast cancer tumours, thus the findings from this study can be extended to breast cancer tumours suggesting that the increased levels of IGF-1 could possibly cause tumour growth by angiogenesis. To further support this, another *in vitro* study conducted on human breast cancer cell lines found that IGF-1 not only promotes growth of new blood vessels but also promotes growth of the tumour and migration, eventually leading to metastasis [53]. Additionally, research into the IGF-1/IGF-1R pathway in breast cancer has identified the IGF-1/IGF-1R/S100A7 pathway as a key factor in tumour-associated angiogenesis within the breast tumour microenvironment, with S100A7, a calcium-binding protein also known as psoriasin, recognised as a novel target of IGF-1 [54]. Using oestrogen receptor (ER)-positive breast cancer cells, CRISPR-engineered IGF-1R knockout cells, and S100A7-transduced isogenic cells, the study showed that IGF-1/IGF-1R signalling activates STAT3, which binds to the S100A7 promoter, thereby increasing S100A7 expression in breast cancer cells [54]. In human vascular endothelial cells, S100A7 activates receptor for advanced glycation end-products (RAGE) signalling, promoting angiogenesis [54]. Collectively, the findings of these studies provide valuable insights into the molecular mechanisms behind the role of IGF-1 and breast cancer progression and warrant further investigation into the inhibition of IGF-1 in clinical trials.

## Therapeutic implications

Currently, none of the FDA-approved breast cancer treatments actively target the inhibition of any GH or IGF-1 action [55]. However, much of the above research justifies further study and investigation into blocking pathways of GH and IGF-1 that promote metastasis and angiogenesis.

As of today, there is one approved GHR antagonist, pegvisomant, established in 2003 and used to treat GH-related disorders such as acromegaly – a rare hormonal disorder where patients produce GH in excess [56]. In a unique case study of a male patient who was diagnosed with acromegaly and later, breast cancer, researchers found that discontinuing pegvisomant therapy resulted in elevated levels of IGF-1 in the patient’s serum which subsequently resulted in breast cancer progression and metastasis to the lungs [56]. Upon resuming pegvisomant treatment alongside treatment for breast cancer (tamoxifen), a metastatic reduction was detected, and IGF-1 levels were normalised. Thus the researchers reported a successful method of treating acromegaly to control breast tumour progression in a male patient [56].

A potential issue with applying these findings to more common cases of breast cancer is the difference in sex between the majority of breast cancer sufferers and this patient. Therefore, extrapolating the data gained in this study to more common cases of breast cancer may not be possible unless the exact mechanisms behind GH and breast cancer are fully understood. These data emphasise the need to uncover the molecular relationship between GH–IGF-1 axis and breast cancer and highlights the potential of combination therapies to treat the two conditions.

Beyond direct GHR inhibition, several upstream inhibitory strategies targeting GH secretion have also shown therapeutic promise. Synthetic somatostatin analogues (SSAs) – including second-generation agents such as pasireotide and paltusotine – reduce pituitary GH release by activating somatostatin receptors (SSTR1–SSTR5), with SSTR2 and SSTR5 being the dominant subtypes in endocrine tissues [57,58].

Pasireotide, originally developed for acromegaly, has been evaluated for its potential to suppress IGF-1–driven pre-malignant progression in the mammary gland. In a short-term study involving 13 women with atypical hyperplasia (AH) in breast, pasireotide significantly reduced proliferation and increased apoptosis in all AH, accompanied by decreased phosphorylation of IGF-1R, ERK1/2 and AKT, with no detectable effect on oestrogen receptor (ER) or progesterone receptor (PR) expression [59]. These molecular changes align with the established GH/GHR-dependent activation of MEK and PI3K pathways in breast tumour models. However, hyperglycaemia – arising from SSA-induced reductions in insulin secretion – remains a notable adverse effect and may limit broader clinical application [59,60]. Moreover, the short treatment window and small cohort size highlight the need for larger, controlled studies to determine whether SSAs can meaningfully influence early breast cancer progression. Subsequently, mechanistically informed trials involving patients with different subtypes of breast cancer are warranted to determine whether therapeutically dampening the GH/GHR axis may offer a rational strategy and can be integrated into breast cancer prevention or treatment paradigms.

## Conclusion

In conclusion, clinical and experimental evidence supports the implications of growth hormone and its receptor in the initiation, progression and spread of breast cancer through its effects on cellular proliferation, tumour growth and survival. The inhibition of GHR holds potential for improving breast cancer prognosis. Future research should focus its efforts in identifying novel therapeutics for the treatment of this disease by targeting GH expression and signalling. In order to successfully develop these targeted therapies aimed at disrupting GH signalling pathways, the understanding of the molecular mechanisms of GH in breast cancer is essential. The use of animal studies has been imperative in gaining an understanding of the action of GH/GHR and IGF-1 in breast cancer cell lines, and this knowledge will hopefully inform future studies and, if relevant, take its place alongside current breast cancer treatments *in vivo* and in pre-clinical and clinical trials to attempt to alleviate the burden of breast cancer.

## Data Availability

This is a review article so no data sharing is required.

## Abbreviations

AH: atypical hyperplasia
EMT: epithelial-to-mesenchymal transition
GH: growth hormone
IGF-1: insulin-like growth factor-1
NRF2: nuclear factor erythroid 2-related factor 2
SSA: synthetic somatostatin analogue
TNBC: triple-negative breast cancer
VEGF: vascular endothelial growth factor
